# Supplementary material for: Real time unsupervised learning of visual stimuli in neuromorphic VLSI systems
Source: Sci Rep. 2015 Oct 14;5:14730. doi: 10.1038/srep14730 (PMC4604465; doi:10.1038/srep14730)
Supplement: Supplementary Information [file srep14730-s1.pdf]

Supplementary Information: Real time unsupervised learning of visual stimuli in neuromorphic VLSI systems

Massimiliano Giulioni[1], Federico Corradi[1,2], Vittorio Dante[1], and Paolo del Giudice[1]

[1]Department of Technologies and Health, Istituto Superiore di Sanitá, Roma, Italy
[2]Institute of Neuroinformatics, University of Zürich and ETH Zürich, Switzerland

July 20, 2015

1 Signal flow

The silicon retina [1] is a detector of dynamic contrast: it continuously measures at every pixel location changes in luminosity contrast and encodes them into output firing rate of the its corresponding neurons. It is frameless and asynchronous: it does not sample frames, but outputs digital event asynchronously in time, depending on the local events of changing contrast, and it uses for this the AER bus, a de facto standard for the communication infrastructure of neuromorphic devices. A moving object on a static background induces high firing rates for retina neurons affected by the moving contours of the object, and very low rates (noise) for neurons stimulated by the static background. There are two neurons associated to each pixel, activated by on-off and off-on luminosity transitions respectively. In our experiments we lump together the two, and only use information on the transition event. The silicon retina is positioned in front of an LCD screen that we used to display visual objects. The refresh rate of the LCD screen is $50Hz$. For the purpose of mapping retina's output to the input of the neurons in the recurrent network, the 16k retina pixels have been grouped into a grid of 14x14 macro-pixels, each containing 81 retina pixels; the spike trains generated by all pixels in one macro-pixel converge to 40 synapses on the dendritic tree of a single excitatory neuron on the chip). Retina pixels can be spontaneously active and emit bursts at few hundreds Hertz. Grouping pixels in macro-pixels, besides being necessary to establish a correspondence between retina and neural chips, serves the purpose of averaging out such high-frequency

sparse retina noise (see Fig. 1). The visual object displayed on the screen excites groups of retina pixels slightly smaller than a macro-pixel (see Fig. 1), to allow for small alignment mismatch between retina and LCD screen.

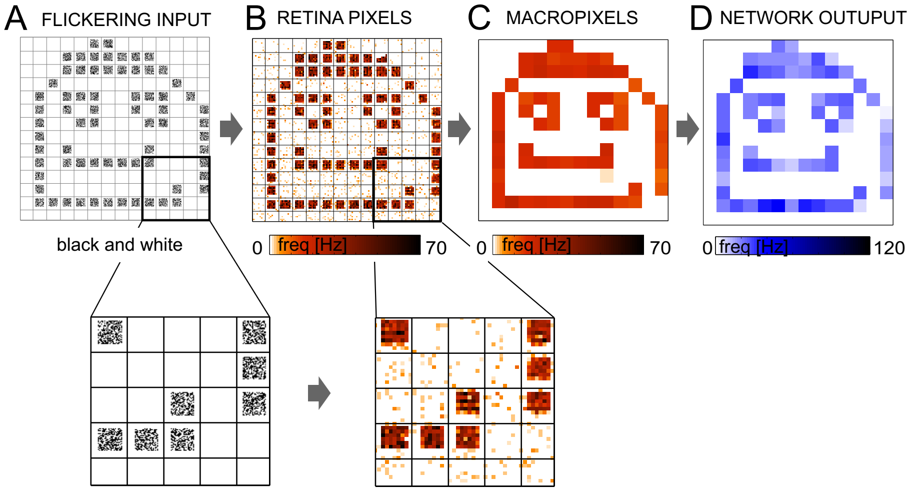

Figure 1: **A** flickering input displayed on the LCD screen in front of the retina. **B** Retina pixel activity accumulated over half a second. Colors encode mean firing rates (Hz). Noisy activity from non-stimulated pixels as well as non-homogeneous activity of stimulated pixels are visible. **C** macro-pixels activity obtained by grouping together the activity of single pixels. It represents the input to the excitatory neurons of the network. **D** Activity induced in the excitatory population of the network.

In order to match the displayed stimuli to the sensitivity of the retina to dynamic contrast, we made them artificially 'dynamic'. Every single screen pixel can be black or white and it is stochastically updated at every LCD frame with a fixed tunable probability of being white: quickly flickering pixels will elicit stronger retina response; tuning such probability allows a good trade-off between a bad retina signal/noise ratio (slow flickering) and fast flickering saturating the retina response, and also loading excessively the AER bus.

2 Theory-inspired tools to control the neuromorphic system

The parameter space of the system is large, and theory should be used to identify regions in such space where the system is able to generate the desired behavior. However, it is often the case that the logically simple route, of tuning biases to precisely match the parameters entering the theoretical model to the corresponding quantities in the circuital embodiment, leads to unsatisfying results or is exceedingly complex. This is so for the many reasons for which the hypothe-

ses underlying the theoretical model do not hold for the silicon network (such as the diffusion approximation). Furthermore, at the lower level, it is clear that the real dynamics of the circuit implementing an element of the model, such as the membrane potential and its dynamics, can only crudely approximate the corresponding equations in the model. It is therefore appealing to use theory as an inspiration to devise a strategy that carries over to the chip, where it is self-consistently applied; in the next section such considerations are made concrete. Achieving such a dynamic 'system identification' of the silicon network, predictive of its response to varying stimuli and needed to control it, is important to bring the neuromorphic system to the level of 'neurophysiology on silicon' we aim at.

2.1 Single neuron transfer function

The first step we take is to characterize the non-linear response of the single silicon neuron, which we do by measuring its 'transfer function', i.e. the output firing rate of the neuron as a function of its input $\nu^{\text{out}} = \phi(\nu^{\text{in}})$. Assuming that the neuron's input is noisy (which is the case because the input from the retina is noisy, because of the quenched noise in the connectivity and because of the circuital mismatches), the output firing of the neuron will not only depend on the average input but also on its fluctuation, such that noise-driven firing is possible even when the average input is below threshold. The measured transfer function is shown in Fig. 2. The dependence of the transfer function on the input mean and variance is in qualitative agreement with the theoretical predictions from the diffusion approximation, and it could be controlled so as to ensure a good sensitivity for the relevant dynamic range of the retina activity, and to have neurons' output firing rates compatible with a sensible spike-driven dynamics of plastic synapses. Neural parameters (see Caption of Fig. 2) were chosen by fitting the theoretical expression for ϕ, given the independently measured values of the synaptic efficacies. The absolute refractory period of the neurons have been tuned to 1.57 ± 0.14ms while the value of the constant leakage current has been set to 10.5 ± 2.8 in units of $\theta - H$, where θ is the spiking threshold and H is the reset potential. All the synaptic efficacies and their distribution across the chips have been measured using a protocol described in [2] based on neuronal response to known input stimuli. The dispersion in the values of synaptic efficacies was found to be roughly 50% of their average, due to mismatch in the synaptic circuits. The mapping from macro-pixels to the 40 synapses per neuron was also effective in averaging out such mismatch effects. Synapses from the retina to the excitatory population have efficacy $J_{pot}^{ExcRet} = 0.075 \pm 0.038$. Recurrent depressed synapses within the excitatory population are set to have efficacy $J_{dep}^{ExcExc} = 0$, while the efficacy of potentiated recurrent synapses is $J_{pot}^{ExcExc} = 0.135 \pm 0.071$. Same efficacy for the excitatory synapses connecting the Retina to the inhibitory neurons. Inhibitory connections have a synaptic efficacy $J_{pot}^{InhExc} = 0.108 \pm 0.053$. All the values are given in the form of mean $\pm$ standard deviation.

Figure 2 shows the single-neuron transfer function $\langle\phi(\nu_{in})\rangle$ averaged over 20

neurons. The stimuli for this measure were poisson synthetic spikes trains via the PCI-AER board.

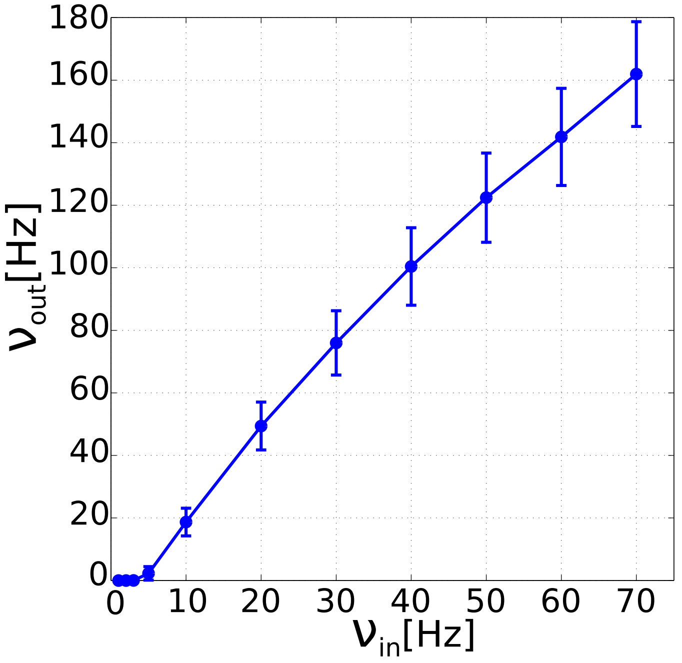

Figure 2: **Single neuron transfer function.** Single-neuron transfer function $\langle \phi(\nu_{in}) \rangle$ averaged over 20 neurons randomly distributed over the two different devices. The input signal is provided by the computer and it consists of a Poisson spike train of mean ν_{in}. Error bars represent the SD of the distributions.

2.2 Feedback gain of the network

The input-output response function of the network will depend on the one of single neurons, and on the massive feedback implemented by the recurrent synapses; since the latter change as learning proceeds, so will the network response function, and it is intuitive that the more learning increases the self-excitability of the network, the more its response function will be non-linear. In the scenario of autonomous learning we focus on, the intertwined dynamics of neurons and plastic synapses can therefore be compactly seen as a dynamic shaping of the network response function as a result of the flow of stimuli.

For fixed strength of the synaptic couplings, in the mean field approximation (i.e. assuming that all neurons in a homogeneous population share the same statistical properties – mean and variance – of their input) the average firing rate of the stationary states of the system are the solution of the self-consistency

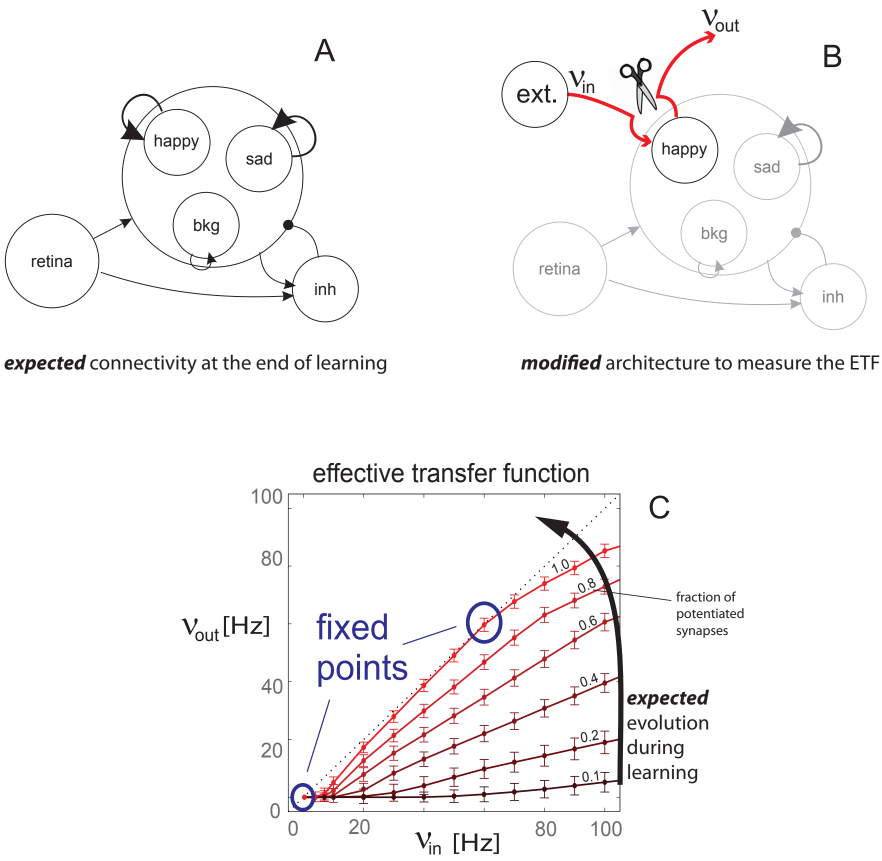

Figure 3: **Effective Transfer Function.** Panel A shows the expected structure of the connectivity at the end of a successful learning trajectory, the thickness of the arrows stands for the strength of the connection. Panel B reports the modified architecture for the measure of the effective transfer function. The population Ext is a replica of the happy population and is simulated on the external PC. Panel C reports the transfer function for increasing level of potentiation in the synapses connecting Ext to happy, thus emulating an increasing level of self-excitation for happy. On the x-axis we plot the mean input frequency ν^{in} exciting the happy population, on the y-axis the average frequency ν^{out} at which the happy relaxes. The intersections between the measured curves and the line $\nu^{in} = \nu^{out}$ are the predicted fixed points of the network dynamics.

equations [3]: $\vec{\nu} = \vec{\phi}(\vec{\nu}, \vec{\nu}^{\rm stim})$ (where the elements of $\vec{\nu}$ are the average firing rates of all the interacting populations, and $\nu_i^{\rm stim}$ is the firing rate encoding the visual stimulus and affecting population i). The solution(s) of the mean field equations identify global, simultaneously stable, state(s) of equilibrium

of the network, in which the firing rates of the different neural populations fluctuate (because of finite-size effects) around the found solutions; external stimuli (or fluctuation) can provoke transitions between such states, which are then stimulus-dependent attractors of the dynamics (i.e. each one of them is the end-point of the dynamics, up to fluctuations, for initial conditions close enough to it).

In general the network will include several interacting populations, and getting insight into the dependence of the mean field solutions on the relevant parameters is in general difficult; one convenient approach is to devise an effective, low-dimensional representation of the mean field vector equation, as was done in [4].

In this approach (assuming for example that we seek a 1-dimensional mean field representation) the mean-field solutions for a multi-population network are found with the firing rate of one population i of interest held fixed at ν_i^{in}, and the stationary states for other populations are found, driving in turn the population of interest to a new average firing rate ν_i^{out}; the procedure is iterated sweeping values of ν_i^{in}, and the result is a one-dimensional $\nu^{\mathrm{out}} = \nu^{\mathrm{out}}(\nu^{\mathrm{in}}, \nu^{\mathrm{stim}})$: the Effective Transfer Function, ETF representation of the transfer function for the population of interest, taking into account its interaction with all the others. The method predicts for the firing rate of the stationary states the same values that would be obtained by directing solving the vector mean field equation, while it offers an approximate description of the (mean field) relaxation dynamics to the stable stationary states, the quality of which depends on the typical time scales of the dynamics for the different neural populations.

Consistently with our approach discussed above, we do not strive to obtain a good quantitative match between mean-field calculations and the chip behaviour, since it would be subject to several unwarranted assumptions. Rather, we introduced in [5] a procedure to directly implement on chip an estimate of the ETF, which is an on-chip embodiment of the iterative mean field procedure just described.

Panel A of Fig. 3 shows the pattern of connectivity expected in the network at the end of learning: through learning the initial homogeneous excitatory population of neurons is expected to split into three sub-populations, one reacting to the input visual stimulus "happy", one selective to "sad" and one not reacting to any inputs, we named it the background ("bkg") sub-population. Since we know a-priori the shape of the visual stimuli and the topology of the connectivity from the retina to the excitatory neurons, we can approximately predict the number of neurons that will be part of every sub-population. We note here that this knowledge does not guarantee a successful learning trajectory leading to a robust associative memory. To tune the synaptic parameters such that a successful learning would be possible, we measured, before learning, the ETF for the "happy" sub-population using the architecture depicted in Fig. 3, panel B. During the measurement the synaptic plasticity is disabled and we impose by hand which portion of the synapses is potentiated. To measure the ETF for the "happy" sub-population we cut all the excitatory-to-excitatory recurrent synapses (note the scissors in the figure) by configuring them as 'AER'

synapses accepting input from an external virtual replica of "happy" itself. All the other synaptic connections are left unmodified. The virtual replica is defined on the PC controlling the PCI-AER board [6]; for each virtual neuron, the excitatory AER synapses to the neurons of the "happy" sub-population on chip duplicate the original recurrent synapses.

Synthetic spike trains with a prescribed firing rate are input from the virtual neurons to the chip through the Sequencer of the PCI-AER board, thus provoking the whole network on chip to settle to a new equilibrium state. A range of input firing rates ($\nu^{\rm in}$) on the population in focus is covered; for each of them the firing rate ($\nu^{\rm out}$) to which the population in focus relaxes as a result of the interaction with the other populations is recorded, thus constructing the estimated ETF.

Fig. 3, panel C, illustrates the result: The ETF is plotted for one excitatory sub-population, for different values of the average strength of the feedback excitatory synapses (i.e. for different fraction of potentiated synapses); the firing rates of stationary states are given by the intersection between the ETF and the $\nu^{\rm in} = \nu^{\rm out}$ dotted line: either one stable solution or two stable solutions. It is seen that, as expected from the theory, the non-linearity of the ETF measured on chip increases as self-excitation increases, up to the point where there is an abrupt transition between one to two stable stationary states (a bifurcation in the corresponding deterministic dynamical system). The sequence of ETF curves reported in figure can be seen as a qualitative anticipation of the expected shaping of the network transfer function during learning, as it induces stimulus-selective synaptic strengthening.

3 synaptic model

The synaptic circuit was designed after [7], as described in [2]. The instantaneous state of the synapses is defined by an internal continuous variable $X(t)$, which is subject to change upon the arrival of a pre-synaptic spike, depending on the current value of the post-synaptic membrane potential V: X undergoes an upward (downward) jump J_+ (J_-) if V is found to be above (below) a threshold θ_v; between subsequent pre-synaptic spikes $X(t)$ drifts towards an upper or lower bound (chosen to be 1 and 0 respectively) depending on its value being above or below a threshold θ_x. The synaptic efficacy is determined at any time by the condition $X(t) > \theta_x \rightarrow J = J_{\rm pot}$ - $X(t) < \theta_x \rightarrow J = J_{\rm dep}$. Changes in the synaptic efficacies then occur whenever $X(t)$ crosses the threshold θ_x.

$$X(t) \rightarrow X(t) + J_+ \quad if \quad V_{post} > \theta_v \tag{1}$$

$$X(t) \rightarrow X(t) - J_- \quad if \quad V_{post} \leq \theta_v \tag{2}$$

J_+, J_- are positive constants. A drift terms drives $X(t)$ toward 0 or 1, depending whether $X(t)$ is above or below θ_x:

$$\frac{dX(t)}{dt} = \alpha_{up} \quad if \quad X(t) > \theta_x \tag{3}$$

$$\frac{dX(t)}{dt} = -\alpha_{dw} \quad if \quad X(t) \leq \theta_x \tag{4}$$

References

[1] Lichtsteiner, P., Posch, C., Delbruck, T. A 128x128, 120 dB 15 micro s Latency Asynchronous Temporal Contrast Vision Sensor. IEEE Journal of Solid State Circuits (43):566-576 (2008).

[2] Giulioni, M., Pannunzi, M., Badoni, D., Dante, V., Del Giudice, P. Classification of correlated patterns with a configurable analog VLSI neural network of spiking neurons and self-regulating plastic synapses. Neural Comput. 21(11):3106-3129, (2009).

[3] Renart, A., Brunel, N., Wang, X-J. Mean-Field Theory of Irregularly Spiking Neuronal Populations and Working Memory in Recurrent Networks, in Computational neuroscience: A comprehensive approach 431-490 (2004).

[4] Mascaro, M., Amit, D.J. Effective neural response function for collective population states, Network: Computation in Neural Systems 10(4):351-373 (1999).

[5] Giulioni, M. et al. Robust working memory in an asynchronously spiking neural network realized in neuromorphic VLSI. Frontiers in Neuroscience, 5 doi:10.3389/fnins.2011.00149 (2012).

[6] Dante, V., Del Giudice, P., Whatley, A.M., PCI-AER - Hardware and Software for Interfacing to Address-Event Based Neuromorphic Systems, The Neuromorphic Engineer 2:5-6 (2005).

[7] Fusi, S., Annunziato, M., Badoni, D., Salamon, A., Amit, D. J. Spike-driven synaptic plasticity: theory, simulation, VLSI implementation. Neural Comput. 12(10):2227-2258 (2000).
